# Successful ixazomib treatment for relapsed and refractory acute myeloid leukemia transformed from myelodysplastic syndrome

**DOI:** 10.1002/ccr3.4287

**Published:** 2021-06-23

**Authors:** Satoko Oka, Kazuo Ono

**Affiliations:** ^1^ Division of Hematology Japanese Red Cross Society Wakayama Medical Center Wakayama Japan; ^2^ Division of Pathology Japanese Red Cross Society Wakayama Medical Center Wakayama Japan

**Keywords:** acute myelogenous leukemia, ixazomib, Myelodysplastic syndrome, NF‐κB

## Abstract

Elevated NF‐kB levels have been identified in primitive bone marrow cells from patients with MDS/AML, suggesting NF‐kB as a therapeutic target in MDS/AML. We herein describe an MDS patient ineligible for SCT who, following treatment with azacitidine and bortezomib, transformed to leukemia, but maintained complete remission after monotherapy with ixazomib.

## Graphical abstract

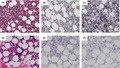

## 1 INTRODUCTION

Elevated NF‐kB levels have been identified in primitive bone marrow cells from patients with MDS/AML, suggesting NF‐kB as a therapeutic target in MDS/AML. We herein describe an MDS patient ineligible for SCT who, following treatment with azacitidine and bortezomib, transformed to leukemia, but maintained complete remission after monotherapy with ixazomib.

Myelodysplastic syndrome (MDS) is a hematopoietic stem cell disorder that is characterized by ineffective hematopoiesis and often transforms into acute myeloid leukemia (AML). Although treatments for MDS/AML have advanced, the treatment for elderly patients who are not eligible for SCT remains inadequate. Thus, novel pharmacologic therapies capable of preventing or delaying disease progression are in critical need. Elevated nuclear NF‐kB levels have been identified in primitive bone marrow cells from patients with MDS, suggesting NF‐kB as a therapeutic target in MDS.^1^ Recent reports showed that bortezomib, a potent inhibitor of the proteasome and NF‐kB, was effective as MDS/AML treatment.^2^, ^3^, ^4^, ^5^ Ixazomib has a shorter proteasome dissociation half‐life and improved pharmacokinetics, and has a low rate of peripheral neuropathy.^6^ Moreover, it inhibits cell growth and induces apoptosis in myeloma cells resistant to bortezomib.

We herein a report of successful treatment with ixazomib in a patient with MDS transformed to AML who previously failed to respond to azacytidine (AZA) and bortezomib treatment.

## 2 CASE REPORT

In 2016, a 78‐year‐old female was referred to our hospital with leukopenia (white blood count 1.8 × 10^9^/L with 3% of peripheral blast cells), anemia (hemoglobin 64 g/L), and hyperproteinemia (total protein 11.0 g/dL). Her renal function and levels of calcium, serum albumin, and lactate dehydrogenase were normal. The serum β2‐microglobulin level was 4.0 mg/dL, and the concentrations of IgG and κ‐light chain were 28.8 g/L (normal range, 8.7‐17 g/L) and 147 mg/dL (normal range, 3.3‐19.4 mg/dL), respectively. The serum free light chain ratio was 4.9 (normal range: 0.26‐1.25). Serum protein electrophoresis disclosed a monoclonal spike in the γ‐globulin region, and urine electrophoresis also revealed a monoclonal spike. Serum immunofixation electrophoresis confirmed the presence of an IgG‐κ chain monoclonal M component. Bone marrow examination showed that 18% were blasts with dysplasia of erythropoiesis, granulopoiesis, and megakaryopoiesis and flow cytometric analysis was positive for CD13, CD33, CD34, and HLA‐DR. Moreover, bone marrow aspiration showed 13% plasma cells and flow cytometric analysis was positive for CD38, CD138 and kappa, and negative for CD19, CD20, and lambda. Strong nuclear CD34 and p53 immunostaining was detected in 8% and 10% of hematopoietic cells, respectively, and CD138 immunostaining was noted in 5% of plasma cells. Karyotype analysis indicated a 46, XX karyotype (20/20 cells). Interphase fluorescence chromosomal in situ hybridization of the bone marrow cells revealed no gene abnormalities in 1q21, RB1, P53, D13S319, and IgH. She had no evidence of myeloma‐defining events or amyloidosis. A diagnosis of the simultaneous occurrence of MDS with excess blast‐2 (International Prognostic Scoring System: high) and monoclonal gammopathy of undetermined significance (MGUS) was made, and 5‐azacytidine (AZA) (75 mg/m^2^, days 1‐7 in a 28‐day cycle) treatment was initiated. After 2 cycles of this treatment, she had normal peripheral blood counts, and serum IgG paraprotein and β2‐microglobulin (β2‐MG) levels decreased to 16 g/L and 1.8 mg/L, respectively. A repeat marrow examination showed slightly hypercellular marrow, with a significant decrease in myeloblasts to 1% blasts and 5% plasma cells. Strong nuclear CD34 and p53 immunostaining decreased in hematopoietic cells and CD138 immunostaining was detected in a few plasma cells. She achieved hematological CR and continued AZA treatment.

In September 2018, after 30 cycles of AZA, the patient exhibited evidence of disease progression, with leukopenia (white blood count 2.1 × 10^9^/L with 1% of peripheral blast cells), anemia (hemoglobin 56 g/L), and hyperproteinemia (total protein 10 g/dL). Serum IgG paraprotein and β2‐microglobulin (β2‐MG) levels increased to 42 g/L and 4.0 mg/L, respectively. The serum free light chain ratio was 7.7. Marrow examination showed slightly hypercellular marrow, with 7% blasts and 8% plasma cells. Strong nuclear CD34 and p53 immunostaining was detected in 5% and 10% of hematopoietic cells, respectively, and CD138 immunostaining was noted in 20% of plasma cells. Karyotypic analyses indicated a 46, XX karyotype (20/20 cells). She was administered bortezomib and dexamethasone (VD) (bortezomib, 1.3 mg/m^2^, days 1, 4, 8, and 11; dexamethasone, 40 mg/day, days 1‐4). After 3 cycles of the treatment, she achieved hematological CR, and her serum and urine M protein were negative by immunofixation, and bone marrow examination showed a decrease in plasma cells (4%). She continued the VD treatment.

**FIGURE 1:**
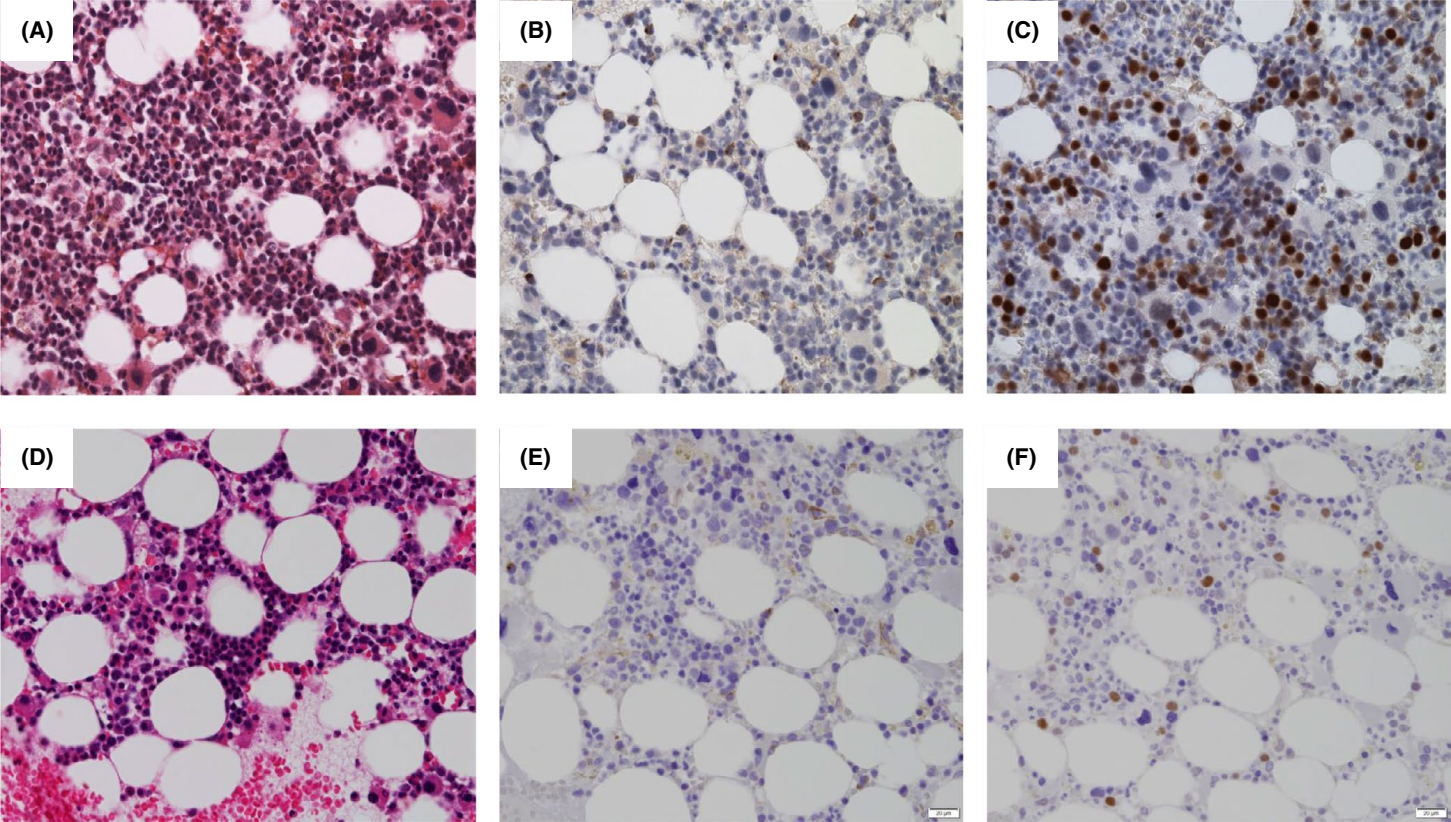
Bone marrow biopsies of acute myeloid leukemia from myelodysplastic syndrome with MGUS (A, B, C) and after ixazomib treatment (D, E, F). A, Hypercellular marrow with myelodysplasia‐related changes and numerous blasts. B, CD34 immunostaining in 10% of blast cells. C, Strong nuclear p53 immunostaining in numerous hematopoietic cells. D, Hypercellular marrow with myelodysplasia‐related changes and decreased blasts. E, CD34 immunostaining in blast cells. F, Strong nuclear p53 immunostaining in a few hematopoietic cells

However, in June 2019, after 10 cycles of VD, the patient exhibited evidence of disease progression, with leukocytosis (WBC 12.5 × 10^9^/L and 5% of blast cells) and anemia (hemoglobin 70 g/L). Serum IgG paraprotein and β2‐microglobulin (β2‐MG) levels increased to 38 g/L and 5.8 mg/L, respectively. The serum free light chain ratio was 5.7. The bone marrow was hypercellular marrow and showed that blasts were 20% and plasma cells were 7% with dysplasia of erythropoiesis, granulopoiesis, and megakaryopoiesis (Figure 1A). Strong nuclear CD34 and p53 immunostaining was detected in 10% and 30% of hematopoietic cells, respectively, and CD138 immunostaining was noted in 8% of plasma cells (Figure 1B and C). Karyotypic analyses indicated a 46, XX karyotype (20/20 cells). The patient was then administered ixazomib and dexamethasone (ixazomib 3 mg/day, days 1, 8, and 15; dexamethasone, 40 mg/day, days 1, 8, 15, and 22). Grade 2 hematologic toxicity was observed. After 1 cycle of this treatment, she had normal peripheral blood counts, and serum IgG paraprotein and β2‐microglobulin (β2‐MG) levels decreased to 28 g/L and 2.2 mg/L, respectively. Repeat marrow examination showed slightly hypercellular marrow, with a significant decrease in myeloblasts to 1% blasts and 2% plasma cells (Figure 1D). Strong nuclear CD34 and p53 immunostaining decreased in hematopoietic cells, and CD138 immunostaining was detected in a few plasma cells (Figure 1E and F). The ixazomib and dexamethasone treatment was continued and the patient maintained CR without progression of AML and MM in the subsequent 21 months.

## 3 DISCUSSION

The standard chemotherapy for MDS/AML achieves remission, although most patients will relapse within 3 years.^7^ In spite of salvage options, including advanced chemotherapy and allogenic SCT, the prognoses of patients who relapse are uniformly poor.^7^ In elderly patients, the prognoses for primary and relapsed MDS/AML are even worse. The hypomethylating agent, AZA, is an effective therapy not only for low‐risk MDS, but also for high‐risk MDS and oligoblastic AML.^8^ However, few effective therapies are available for elderly MDS patients, especially after the failure of hypomethylating agents. Thus, there is a clear and emerging need for the development of new therapeutic approaches.

One potential molecular approach is to target nuclear factor‐kappa B (NF‐kB), a master transcription factor involved in cell growth and proliferation. Elevated nuclear NF‐kB levels have been identified in primitive bone marrow cells from patients with MDS/AML, suggesting NF‐kB as a therapeutic target in MDS/AML.^1^ Proteasome inhibitors, such as bortezomib, carfilzomib, and ixazomib, are now incorporated into the standard of care regimens for most patients with MM and other plasma cell disorders, and this approach has yielded significantly improved clinical responses and overall survival for these patients.^9^ These agents are thought to have inhibitory activity against NF‐κB. Indeed, bortezomib induces apoptosis in vitro in bone marrow cells from individuals with MDS and AML, and treatment with bortezomib showed effective clinical responses in MDS patients.^2^, ^3^, ^4^, ^5^ According to previous studies, the addition of bortezomib to daunorubicine/cytarabine or MEC (mitoxantrone, etoposide, and cytarabine) in patients with relapsed/refractory AML showed improvements in response.^10^, ^11^ Moreover, bortezomib benefits patients who have previously failed from treatment with hypomethylating agents. A previous study demonstrated that bortezomib has hypomethylating activity via down‐regulation of DNA methyltransferase expression, rather than by direct enzymatic inhibition,^12^ which may partly explain the activity of bortezomib even after resistance to hypomethylating agents.

Ixazomib, the biologically active boronic acid form of ixazomib citrate that forms immediately upon exposure to aqueous solution or plasma, has a proteasome dissociation half‐life that is sixfold faster than that of bortezomib.^5^ Additionally, ixazomib inhibits cell growth and induces apoptosis in MM cells resistant to conventional therapies and bortezomib. This rapid‐on and rapid‐off pharmacodynamics makes this drug potentially more specific for tumor cells and may lead to an improved therapeutic index.^13^ A recent study showed that ixazomib induces reactive oxygen species and has some antileukemic activity in human AML cells.^14^ Advani et al demonstrated that the regimen of MEC and ixazomib was well tolerated and associated with a high clinical response rate for relapsed/refractory AML patients.^5^ Its substantial oral bioavailability and weekly or twice‐weekly scheduling are additional attractive properties for diseases such as MDS/AML, which primarily affect elderly populations that may not tolerate intensive treatment regimens. Furthermore, ixazomib has significantly less neurotoxicity than bortezomib and has displayed a manageable safety profile during early‐phase clinical trials in refractory and heavily pretreated myeloma populations.^15^, ^16^ This improved tolerability profile places ixazomib as a promising antileukemic agent for maintenance therapy or for combination therapy with other antileukemic drugs in the treatment of AML. The present case showed that monotherapy with ixazomib 3 mg is effective and tolerable for an elderly transformed to AML patient with previous AZA and bortezomib treatment failure.

The frequencies of TP53 mutations in MDS and MM were 7‐19 and 8%‐15%, respectively, and TP53 mutant clones may drive disease progression.^17^, ^18^, ^19^, ^20^ In MDS, p53 nuclear expression has been correlated with hemizygous TP53 mutations and strong p53 immunostaining in >1% of bone marrow progenitor cells has been correlated with a higher risk of AML.^17^, ^18^ In MM, the presence of TP53 mutations indicates a dismal prognosis, similar to MDS; patients exhibiting a more aggressive disease course more frequently have extramedullary disease and hypercalcemia, as well as shorter overall and progression‐free survival.^19^, ^20^ In the present case, the percentage of strongly p53‐positive bone marrow cells was >10% at the time of the leukemic transformation and progression to MM; however, this percentage decreased after treatment with ixazomib.

To the best our knowledge, this is the first case report to document successful treatment with ixazomib alone as salvage therapy in an elderly relapse/refractory MDS patient. Although treatments for MDS/AML have advanced, the treatment for elderly patients who are not eligible for SCT remains inadequate. The mechanism of ixazomib action in AML/MDS is still unclear and further investigation is needed.

## CONFLICT OF INTEREST

None declared.

## AUTHOR CONTRIBUTIONS

SO: was involved in patient care and writing the manuscript. KO: has reviewed and arranged the pathology images.

## ETHICAL APPROVAL

Informed consent was obtained from the patient for publication of this case report and the accompanying images.

## DATA AVAILABILITY STATEMENT

Bone marrow biopsies showed hypercellular marrow with myelodysplasia‐related changes and numerous blasts (A) and strong nuclear CD34 and p53 immunostaining was detected in 10% and 30% of hematopoietic cells (B and C). ​After ixazomib treatment, repeat marrow examination showed slightly hypercellular marrow, with a significant decrease in myeloblasts to 1% blasts and 2% plasma cells (Figure D). Strong nuclear CD34 and p53 immunostaining decreased in hematopoietic cells (Figure E and F).
